# PTP1B Inhibitors from the Entomogenous Fungi *Isaria fumosorosea*

**DOI:** 10.3390/molecules22122058

**Published:** 2017-11-24

**Authors:** Jun Zhang, Lin-Lin Meng, Jing-Jing Wei, Peng Fan, Sha-Sha Liu, Wei-Yu Yuan, You-Xing Zhao, Du-Qiang Luo

**Affiliations:** 1 College of Life Science, Key Laboratory of Medicinal Chemistry and Molecular Diagnosis of Ministry of Education, Hebei University, Baoding 071002, China; zhangjun49@126.com (J.Z.); MLL1122334@126.com (L.-L.M.); fp_neal@126.com (P.F.); liushasha981@126.com (S.-S.L.); yuanweiyv@126.com (W.-Y.Y.); 2 College of Pharmaceutical Science, Key Laboratory of Pharmaceutical Quality Control of Hebei Province, Hebei University, Baoding 071002, China; wjj921121@163.com; 3 Institute of Tropical Bioscience and Biotechnology, Chinese Academy of Tropical Agricultural Sciences, Haikou 571101, China

**Keywords:** *Isaria fumosorosea*, PTP1B, pyrrolidinedione

## Abstract

Protein tyrosine phosphatase 1B (PTP1B) is implicated as a negative regulator of insulin receptor (IR) signaling and a potential drug target for the treatment of type II diabetes and other associated metabolic syndromes. Thus, small molecule inhibitors of PTP1B can be considered as an attractive approach for the design of new therapeutic agents of type II diabetes and cancer diseases. In a continuing search for new PTP1B inhibitors, a new tetramic acid possessing a rare pyrrolidinedione skeleton named fumosorinone A (**1**), together with five known ones **2**–**6** were isolated from the entomogenous fungus *Isaria fumosorosea.* The structures of **2**–**6** were elucidated by extensive spectroscopic analysis. Fumosorinone A (**1**) and beauvericin (**6**) showed significant PTP1B inhibitory activity with IC_50_ value of 3.24 μM and 0.59 μM.

## 1. Introduction

Tyrosine phosphorylation and dephosphorylation are crucial elements in eukaryotic signal transduction. Phosphatases can be subdivided based on structure and substrate specificity into the protein tyrosine phosphatase (PTP) and protein serine/threonine phosphatase (PSP) classes [1]. More recent evidence has suggested protein tyrosine phosphatase 1B (PTP1B) is a major negative regulator of the insulin signaling pathway [2]. As so far, several ‘classical’ protein tyrosine phosphatases are attractive therapeutic targets, including PTP1B for obesity and type II diabetes; as well as SHP2 for cancer and Lyp for rheumatoid arthritis [3]. Despite the research efforts in academia and industry over the past decade, there are very few PTPase inhibitors that have been advanced into clinical trials [4]. Thus, inhibition of PTP1B also can be considered as an attractive approach for the design of new therapeutic agents for the treatment of type II diabetes and for new antitumor drugs. To date, very few inhibitors have been isolated from microorganisms, particularly insect pathogenic fungi [3]. Therefore, insect pathogenic fungi have been considered as an untapped source of small molecules PTP inhibitors. In our previous study, a PTP1B inhibitor fumosorinone has been reported [5], which was found to improve insulin resistance in type II diabetes [6]. As part of our continuing search for bioactive constituents, a PTP1B inhibitory alkaloid fumosorinone A together with another five known constituents **2**–**6** were isolated from *Isaria fumosorosea*. Herein, the isolation, structural elucidation, and PTP1B inhibitory activity evaluation of these compounds are described.

## 2. Results and Discussion

### 2.1. Identification of Compounds ***1**–**6***

**Figure 1:**
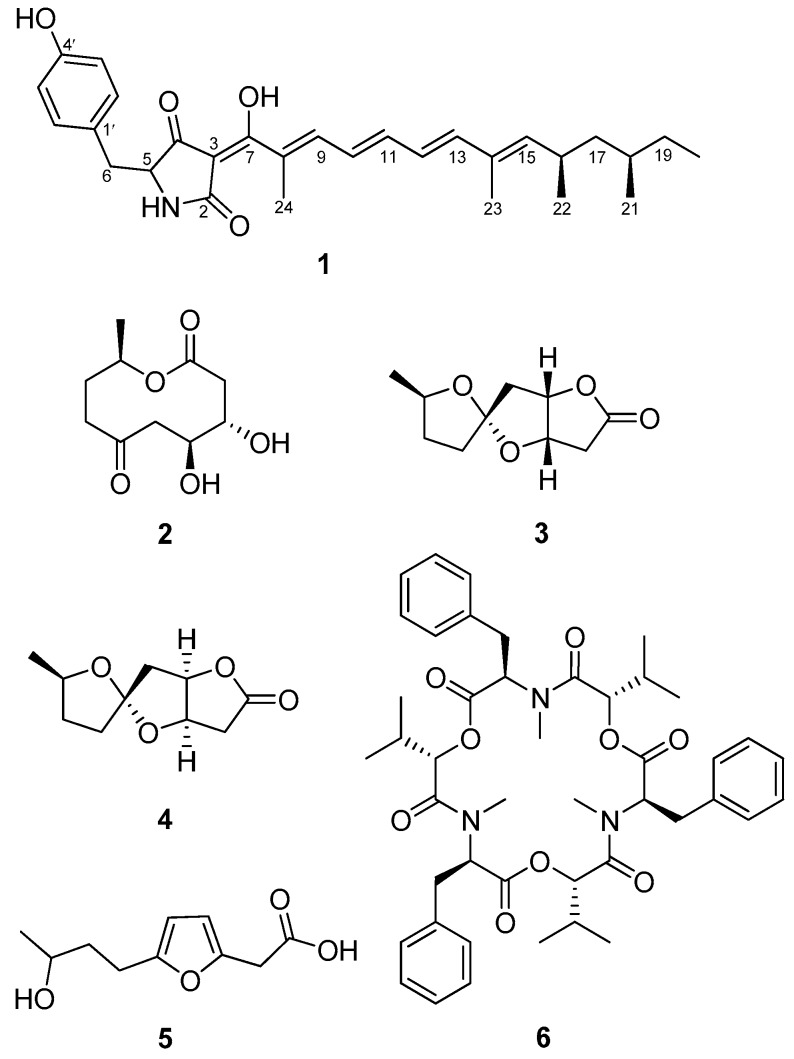
Structures of compounds **1**–**6**.

**Figure 2:**
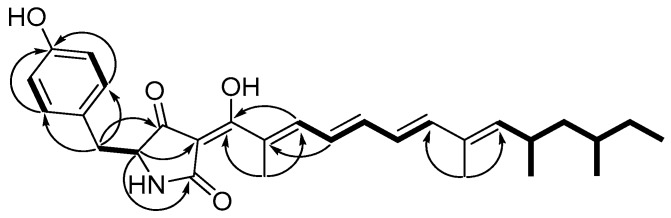
^1^H-^1^H COSY (bold) and key HMBC (arrow) correlations of fumosorinone A (**1**).

Compound **1** was obtained as red oil. It was assigned a molecular formula of C_29_H_37_NO_4_ (12 degrees of unsaturation) by HRESIMS (*m*/*z* 464.2792 [M + H]^+^; calcd for C_29_H_38_NO_4_, 464.2795). The ^13^C-NMR data (Table 1) of **1** along with analysis of the DEPT spectra displayed signals of 5 methyl, 3 methylene, 13 methine, and 8 quaternary carbons (Supplementary Figure S2). The NMR data of **1** were similar to those of militarinone C [7], suggesting that **1** (Figure 1) had a same pyrrolidinedione skeleton deduced from the HMBC correlations (H-5/C-2, C-3, and C-4) and a *p*-hydroxybenzyl group supported by the ^1^H-^1^H COSY cross-peaks (H-2′/H-3′ and H-5′/H-6′) and HMBC correlations (H-6/C-2′, C-6′; H-2′, H-6′/C-4′) (Figure 2). The linkage of the *p*-hydroxybenzyl group with C-5 was established by the ^1^H-^1^H COSY cross-peak of H-5/H-6 and HMBC correlation from H-6 to C-4. The difference between militarinone C and compound **1** was that the side chain of compound **1** had one more propene group than militarinone C. A proton spin system of an olefinic chain, consisting of two doublets (H-9 and H-13) and three doublets of doublets (H-10 to H-12), was readily detected by ^1^H-^1^H COSY signals. The connectivity from C-15 to C-22 was also supported by the ^1^H-^1^H COSY correlations (Figure 2). Two methyls of C-23 and C-24 were unambiguously assigned to C-8 and C-14, respectively, by HMBC correlations from H_3_-24 (*δ*_H_ 2.06, s) to C-7, C-8, and C-9 and from H_3_-23 (*δ*_H_ 1.84, s) to C-13, C-14, and C-15. Therefore, the planar structure of **1** was determined as shown in Figure 1.

The relative configuration of the olefinic moiety in fumosorinone A was determined by analysis of the ^1^H-^1^H coupling constants and NOESY data. The C-10, C-11, and C-12, C-13 double bonds were all assigned E-geometry based on a large coupling constant of 15.2 Hz observed for corresponding olefinic protons, and the same assignment was made for C-14, C-15, and C-8, C-9. The relative configurations of C-16 and C-18 were deduced by the ^13^C-NMR shift values of C-21 and C-22 methyl groups, and a difference of 2.2 ppm revealed their syn-configuration [7,8,9]. Thus, compound **1** was elucidated as shown in Figure 1 and named fumosorinone A.

Compounds **2**–**6** were identified as Cepharosporolides C (**2**), Cepharosporolides E (**3**), Cepharosporolides F (**4**), 2-carboxymethyl-4-(3′-hydroxybutyl)furan (**5**), Beauvericin (**6**), respectively, based on their NMR data and comparison of these data with those reported in the literature [10,11,12].

### 2.2. PTP1B Inhibitory Activity of Compounds ***1**–**6***

Since fumosorinone have been reported to possess PTP1B inhibitory activity [5], compounds **1**–**6** were evaluated for inhibition of PTP1B in vitro. Results indicated that **1** and **6** exhibited significant PTP1B inhibitory activity with IC_50_ value 3.24 and 0.59 μM compared with the positive control Sodium orthovanadate (11.3 μM) (Table 2).

## 3. Materials and Methods

### 3.1. General Experimental Procedures

Optical rotations were measured on a Perkin-Elmer 341 spectropolarimeter (PerkinElmer, Waltham, MA, USA). UV spectra were measured on an UV-210 spectrometer (Analytikjena, Jena, Germany). IR Spectra were acquired on a PerkinElmer 577 instrument (PerkinElmer, Waltham, MA, USA). NMR Spectra were recorded on a Bruker AM-600 spectrometer (Bruker, Fllanden, Switzerland). HR-MS Spectra were recorded on a Bruker apex-ultra 7.0T spectrometer (Bruker, Billerica, MA, USA). Column chromatography (CC) were conducted over silica gel (SiO_2_; 200–300 mesh; Yantai Zhi Fu chemical Co., Yantai, China), and Sephadex LH-20 gel (25–100 μm, GE Healthcare Co., Ltd., Uppsala, Sweden). TLC were conducted with silica gel GF_254_ plates (Yantai Zhi Fu chemical Co., Ltd., Yantai, China).

### 3.2. Fungal Material and Cultivation Conditions

*Isaria fumosorosea* was isolated from an unidentified Lepidopteran collected in Hebei Province, China, and identified by Prof. Yong-Chun Niu, which was assigned the accession number ACCC 37775 in the culture collection at College of Life Science, Hebei University. The fungal strain was cultured on slants of potato dextrose agar (PDA) at 26 °C for seven days, and then inoculated into 500 mL Erlenmeyer flask containing 100 mL of PDA medium (20.0 g of glucose, 200.0 g of potato, 3.0 g of KH_2_PO_4_, 1.5 g of MgSO_4_, 0.1 g of citric acid, and 10.0 mg of thiamin hydrochloride, in 1 L of deionized H_2_O). The final pH of the media was adjusted to 6.5 before sterilization. After seven days of incubation at 26 °C on rotary shakers at 150 rpm, 10 mL of culture liquid were transferred as seed into each 500 mL Erlenmeyer flask containing rice medium (80 g of rice, 100 mL of deionized H_2_O), and the fermentation was carried out at 26 °C under light for 30 days.

### 3.3. Extraction and Isolation

The fermented material was extracted three times with AcOEt (15 L for each time). Evaporation of the solvent in vacuo gave a yellow oily residue (200 g), which was subjected to CC [SiO_2_; petroleum ether (PE)/AcOE 100:0, 95:5, 90:10, 80:20, 60:40, 50:50 (*v*/*v*)] to afford six fractions, Fr. 1–6. Fr. 4 (35 g) was further purified by repeated CC (Sephadex LH-20; Methanol) and prep. TLC [PE/acetone 5:1 (*v*/*v*)] to afford compound **1** (10 mg), compound **2** (15 mg), compound **5** (8 mg), compound **6** (13 mg). Fr. 4 (12 g) was further purified to afford compound **3** (21 mg) and compound **4** (17 mg).

### 3.4. Spectroscopic Data

Fumosorinone A (**1**), C_29_H_37_NO_4_, obtained as red oil; ${\left[\alpha \right]}_{D}^{20}$ −207 (*c* 0.1, MeOH); UV(MeOH) *λ*_max_ (log *ε*): 203 (4.46), 225 (4.40), 307 (4.29) nm; IR (KBr) *λ*_max_ 3395, 2959, 1655, 1515, 1444, 1239 cm^−1^; ^1^H- and ^13^C-NMR data, see Table 1. Positive HR-ESI-MS [M + H]^+^ *m*/*z* 464.2792 (calcd for C_29_H_38_NO_4_ 464.2795).

### 3.5. PTP Assay

PTP1B activity was measured as the rate of hydrolysis of *p*-nitrophenyl phosphate (pNPP) in a 96-well microtiter plate format [13]. Sodium orthovanadate was used as the positive control. Each experiment was performed in triplicate, and IC_50_ data were derived from three independent experiments.

## 4. Conclusions

A new tetramic acid possessing a rare pyrrolidinedione skeleton named fumosorinone A (**1**), along with four known 10-membered macrolides **2**–**5** and a known cyclopeptide **6** identified as cepharosporolides C (**2**), cepharosporolides E (**3**), cepharosporolides F (**4**), 2-carboxymethyl-4-(3′-hydroxybutyl)furan (**5**), and beauvericin (**6**), have been isolated from the an EtOAc extract of entomogenous fungus *Isaria fumosorosea*. Fumosorinone A (**1**) and beauvericin (**6**) showed significant PTP1B inhibitory activity with IC_50_ value of 3.24 μM and 0.59 μM, suggesting novel kinds of protein tyrosine phosphatase inhibitors.

## Figures and Tables

**Table 1:** ^1^H (600 MHz) and ^13^C (150 MHz) NMR data of compound **1** (in CD_3_OD).

No.	*δ*_C_ (Dept)	*δ*_H_ (Mult, *J* in Hz)	No.	*δ*_C_ (Dept)	*δ*_H_ (Mult, *J* in Hz)
2	174.8, C		17a	44.6, CH_2_	1.16 ^a^, m
3	99.7, C		17b		1.34 ^a^, m
4	194.1, C		18	32.3, CH	1.34 ^a^, m
5	61.6, CH	4.05, t (4.8)	19a	29.8, CH_2_	1.16 ^a^, m
6a	36.5, CH_2_	2.89, dd (14.1, 6.1)	19b		1.34 ^a^, m
6b		3.03, dd (14.1, 4.1)	20	10.3, CH_3_	0.88, t (7.4)
7	185.0, C		21	18.2, CH_3_	0.88, d (6.9)
8	128.5, C		22	20.4, CH_3_	0.98, d (6.6)
9	142.7, CH	7.67, d (9.5)	23	11.4, CH_3_	1.84, s
10	126.4, CH	6.70, dd (15.2, 9.5)	24	11.2, CH_3_	2.06, s
11	142.8, CH	6.70, dd (15.2, 9.5)	1′	126.4, C	
12	126.2, CH	6.42, dd (15.2, 9.5)	2′	130.4, CH	7.03, d (8.2)
13	142.8, CH	6.54, d (15.2)	3′	114.7, CH	6.71, d (8.2)
14	132.7, C		4′	155.9, C	
15	143.3, CH	5.45, d (9.8)	5′	114.7, CH	6.71, d (8.2)
16	30.5, CH	2.68, m	6′	130.4, CH	7.03, d (8.2)

^a^ Overlapped signals.

**Table 2:** PTP1B inhibitory activities of **1**–**6**.

Compounds	IC_50_ (μM)
**1**	3.24 ± 0.37
**2**	>1000
**3**	>1000
**4**	>1000
**5**	>1000
**6**	0.59 ± 0.15
Sodium orthovanadate	11.3 ± 0.87

## Supplementary Materials

The 1D- and 2D-NMR spectra are available as Supplementary Materials.
