# Supplementary material for: PTP1B Inhibitors from the Entomogenous Fungi Isaria fumosorosea
Source: Molecules. 2017 Nov 24;22(12):2058. doi: 10.3390/molecules22122058 (PMC6149825; doi:10.3390/molecules22122058)

**Supplementary Materials**

**PTP1B Inhibitors from** **the Entomogenous Fungi *Isaria fumosorosea***

**Jun Zhang ^1^, Lin-Lin Meng ^1^, Jing-Jing Wei ^2^, Peng Fan ^1^, Sha-Sha Liu ^1^, Wei-Yu Yuan ^1^,
You-Xing Zhao ^3^,* and Du-Qiang Luo ^1^,***

^1^ College of Life Science, Key Laboratory of Medicinal Chemistry and Molecular Diagnosis of Ministry of Education, Hebei University, Baoding 071002, China; zhangjun49@126.com (J.Z.); MLL1122334@126.com (L.-L.M.); fp_neal@126.com (P.F.); liushasha981@126.com (S.-S.L.); yuanweiyv@126.com (W.-Y.Y.)

^2^ College of Pharmaceutical Science, Key Laboratory of Pharmaceutical Quality Control of Hebei Province, Hebei University, Baoding 071002, China; wjj921121@163.com

^3^ Institute of Tropical Bioscience and Biotechnology, Chinese Academy of Tropical Agricultural Sciences, Haikou 571101, China

* Correspondence: [zhaoyouxing@itbb.org.cn](mailto:zhaoyouxing@itbb.org.cn) (Y.-X.Z.); [duqiangluo@163.com](mailto:duqiangluo@163.com) (D.-Q.L.)

**Figure S1.** 1H-NMR spectrum of compound **1** (600 MHz, CD3OD).

**Figure S2.** 13C-NMR spectrum of compound **1** (150 MHz, CD3OD).

**Figure S3.** HRMS spectrum of compound **1**.

**Figure S4.** HSQC spectrum of compound **1** (600 MHz, CD3OD).

**Figure S5.** HMBC spectrum of compound **1** (600 MHz, CD3OD).

**Figure S6.** ^1^H-^1^H COSY spectrum of compound **1** (600 MHz, CD3OD).

**Figure S7.** IR spectrum of compound **1**.

**Figure S1**. ^1^H-NMR spectrum of Compound **1**(600 MHz, CD_3_OD) .


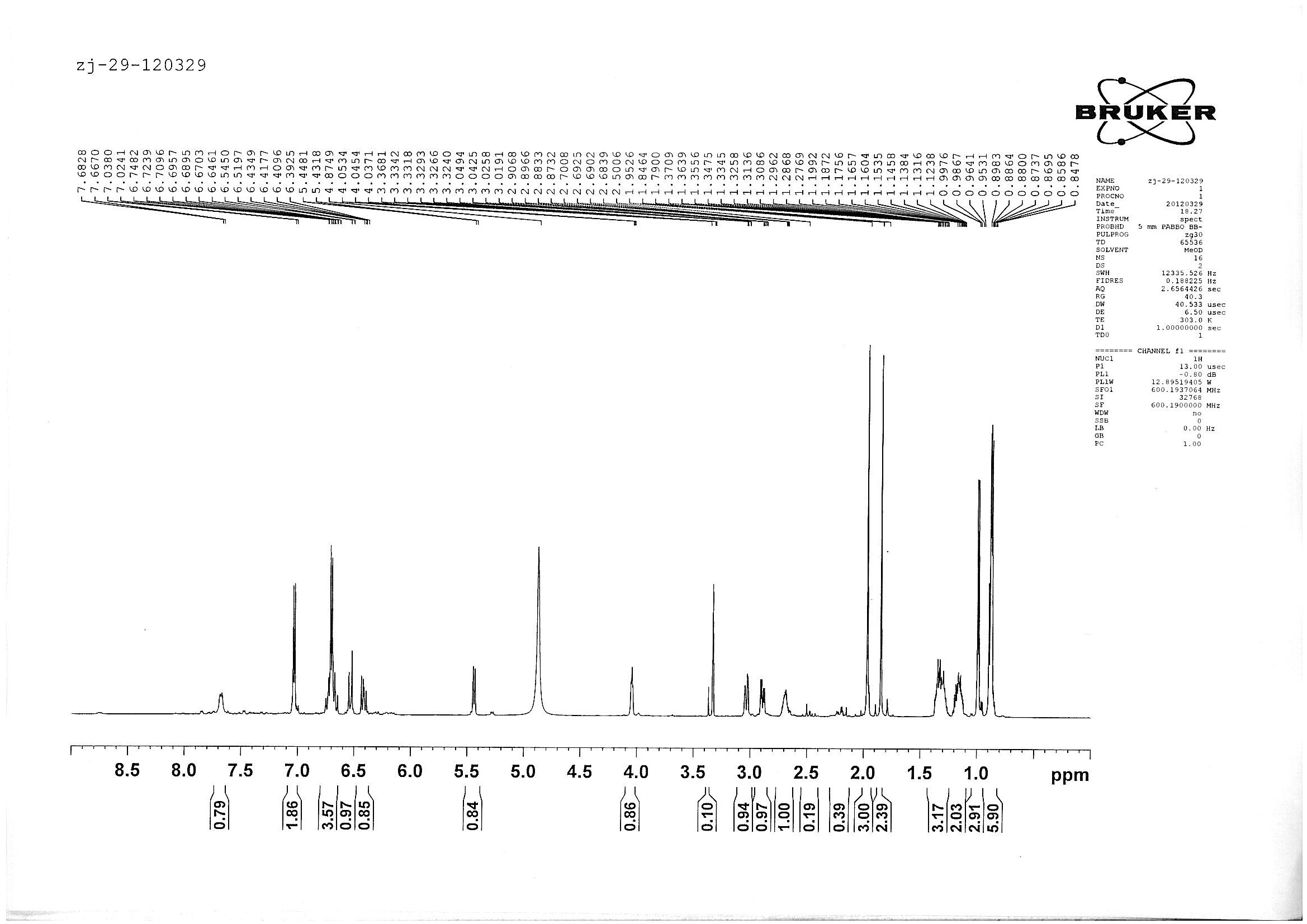


**Figure S2**. ^13^C-NMR spectrum of Compound **1**(150 MHz, CD_3_OD) .


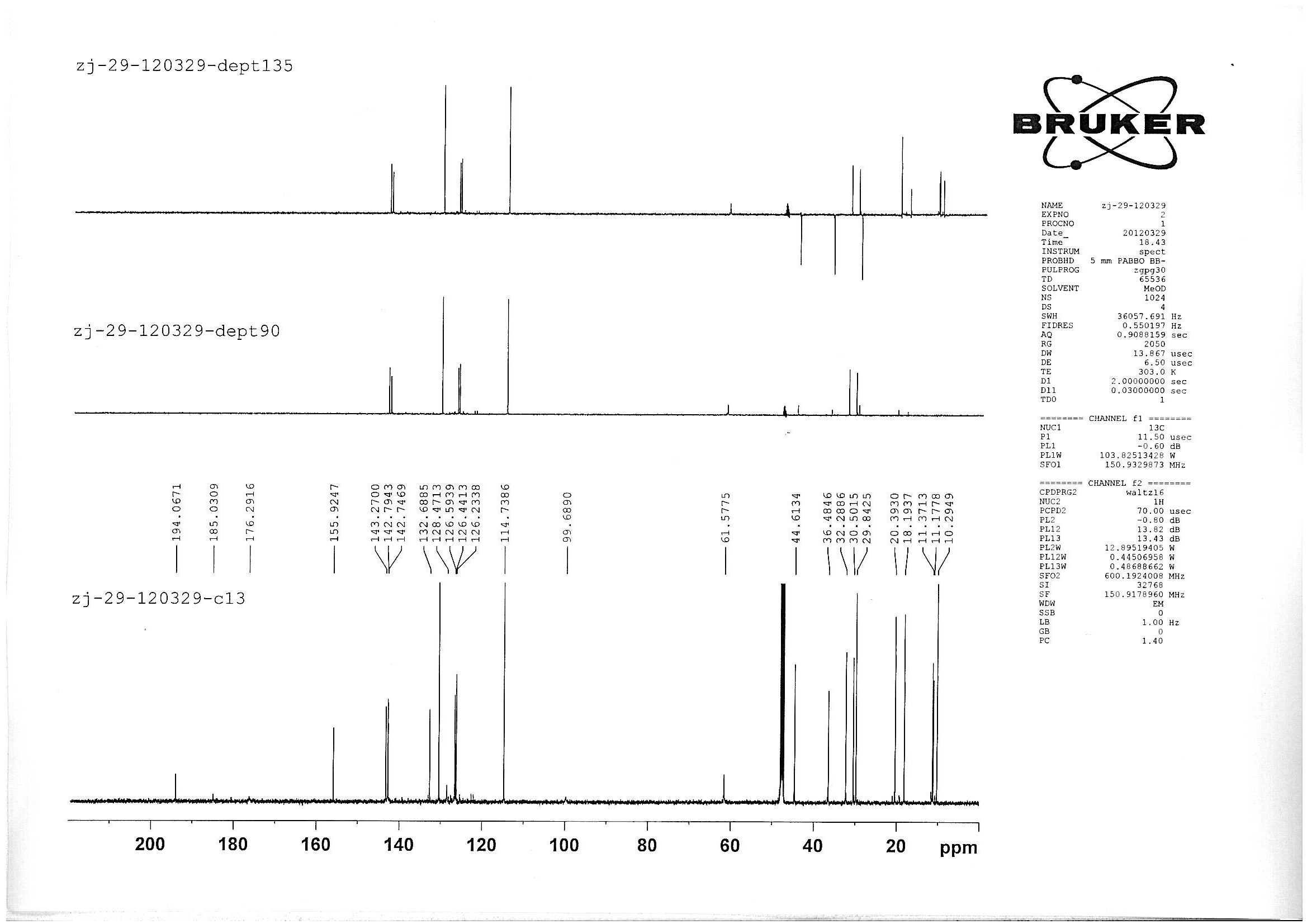


**Figure S3**. HRMS spectrum of Compound **1**.


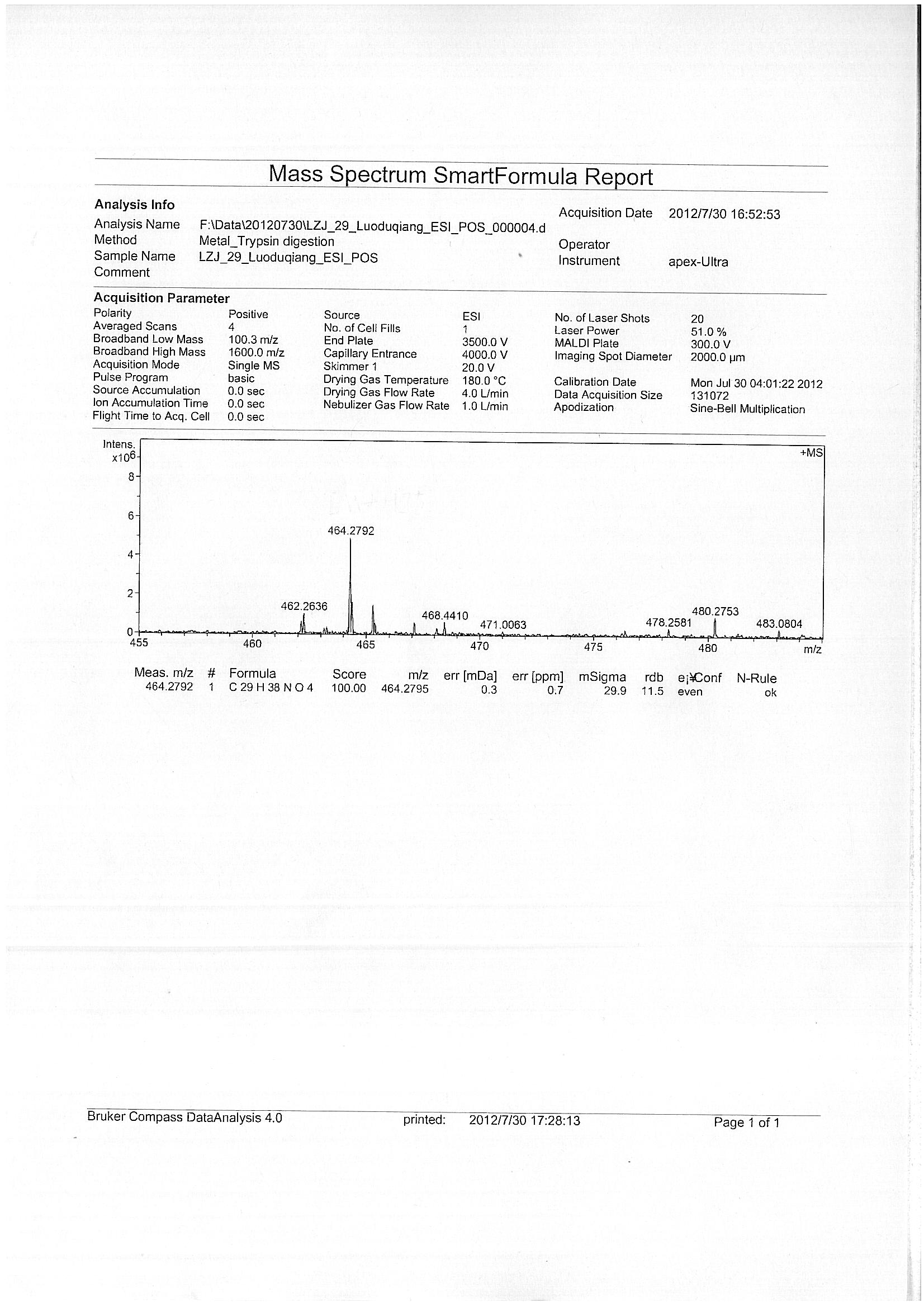


**Figure S4**. HSQC spectrum of compound **1**.

**Figure S5**. HMBC spectrum of compound **1**.

**Figure S6**. H-H COSY spectrum of compound **1**.

**Figure S7**. IR spectrum of compound **1**.


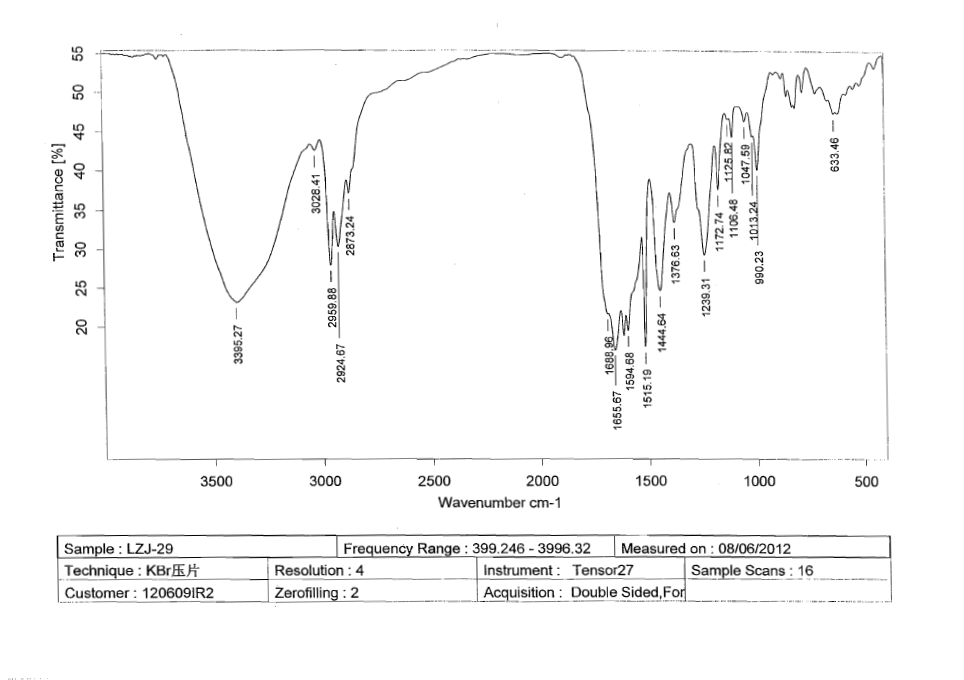

Supplement: Supplementary file 1 [file molecules-22-02058-s001.doc]
